# Analysis of the Sound Environment and the Sound Level in the Delivery Room in the First Hour of a Newborn's Life

**DOI:** 10.34763/jmotherandchild.2020241.1927.000006

**Published:** 2020-07-29

**Authors:** Anna Łozińska-Czerniak, Monika Salamończyk, Ewa Dmoch-Gajzlerska, Magdalena Bednarczyk

**Affiliations:** 1 Department of Obstetrics and Gynecology Didactics, Medical University of Warsaw, Warsaw, Poland; 2 Neonatology and Neonatal Intensive Care Clinic, Institute of Mother and Child, Warsaw, Poland

**Keywords:** delivery room, newborn, noise

## Abstract

**Objective:**

The aim of the study was to analyse the sound environment and the range of sound levels recorded in the delivery room immediately after the birth of a newborn.

**Materials and methods:**

The research method was open observation combined with recording measurements of the sound intensity levels. The material was collected by means of an observation questionnaire. The research was conducted in 11 maternity hospitals in Warsaw. A total of 304 vaginal labours were analysed.

**Results:**

The average sound level in the delivery room after the birth of a newborn was 58.03 ± 7.66 dB, and the sound intensity ranged from 40.30 dB to 78.0 dB. Staff conversations were the most common sources of noise. A statistically significant relationship between the number of people in the delivery room and sound intensity was observed. The number of people positively correlated with the average sound level (R=0.520, p<0.001).

**Conclusions:**

Based on the tests, it was found that the average sound level in the delivery room exceeded the recommended standards. The noise was mainly caused by the activity of staff. The present study indicates the need for staff education and the use of noise reduction procedures.

## Introduction

Sound is the acoustic vibration propagating in an elastic medium, capable of creating auditory sensations that are contained in the band of frequencies between 16 Hz and 20,000 Hz. Sound can be defined as vibration that has two main components: frequency (key), which is measured in hertz (Hz), and intensity (loudness) or sound pressure, usually expressed in decibels (dB) (1). Sounds that are undesirable or harmful to human health, usually of excessive intensity, in a given place and time are called noise. The International Labor Organization defines noise as “any sound that is undesirable, oppressive or even harmful to human health or otherwise dangerous in given conditions” 20 ;,,I(2). According to the recommendations of the Ministry of the Environment in Poland, the permissible noise level in hospitals is 45 dB during the day and 40 dB at night ,,I.(3). The noise level acceptable and recommended in neonatological departments by the American Academy of Pediatrics is 45 dB, while temporary loud sounds not exceeding 65 dB are allowed (4).

The foetus begins to respond to vibroacoustic stimulation at around 24–25 weeks. In the foetal period, what is shaped is not only the reception but also the differentiation of sounds and auditory memory. The foetus is subjected to auditory stimulation in the womb: a continuous, rhythmic mixture of sounds, which consists primarily of the mother's voice. In addition, he/she also receives other stimuli, such as heart tones, breathing murmurs and vibrations caused by the diaphragm. The foetal acoustic environment also consists of sounds caused by low-frequency intestinal peristalsis (5,6). The sounds occurring there have the following features: a continuous, rhythmic and repetitive character; low intensity; and distorted and amortised by e.g. amniotic fluid. They play an important role in the development of hearing and speech after birth, by contributing to the formation of nerve connections in the brain (7). Sounds in the uterus are transmitted to the foetus through the waters to the skull bones. The intrauterine environment is characterised by bone conduction of sounds, and the conduction centre is a fluid that, together with the mother's tissues, suppresses sounds coming from outside. The foetus may be exposed to noise levels >50 dB at low-frequency ranges <500 Hz, with possible peaks >70 dB (8,9), but the barrier consisting of the uterine wall and amniotic fluid can reduce noise by up to 35 dB. The sounds of conversations are received by the foetal hearing organ at about 30% of the original intensity, while intonation and timbre of the voice are well conducted by amniotic fluid (10).

After birth, newborns have no barrier to protect themselves against noise. They are exposed to strange voices, and during this period, they often encounter limited stimulation by the mother's voice. Loud, unpredictable and unorganised sounds prevail, often having a wider frequency spectrum, shifting towards higher intensity compared to the low-frequency sounds heard in the uterus.

In the first hour of life, a newborn baby presents a behavioural condition called alert inactivity, a condition in which he/she is more susceptible to interaction with his/her parents. Inadequate acoustic environment in the delivery room in the form of loud sounds can lead to stress, irritability or discomfort and, as a consequence, impede the first meeting between the parents and the child, which is crucial for the development of bonds (11,12).

Not only does being exposed to excessive noise have a stressful effect on a newborn baby, changing his/her behaviour and affecting development, but it can also cause hearing damage, the type of damage to the hearing organ depending on the frequency, intensity, duration of sound and maturity of the newborn. Undesirable effects of noise depend primarily on its intensity, frequency, the nature of changes over time, duration of action, content of inaudible components and individual characteristics of the recipient. The child's response to these adverse stimuli consists of apnoea, heart rate disturbances, fluctuations in blood pressure, instability of haemoglobin saturation measured by pulse oximetry, hypoxaemia, increased oxygen consumption, secondary increased pulse and respiratory rate, respiratory effort, increased stress hormone secretion, increased glucose levels, increased intestinal peristaltic movements, increased muscle tone, impaired immune function, hypoxia, bleeding into the central nervous system and decrease in the calories needed for growth. Behavioural changes appear in the form of sleep disturbance, agitation, crying, yawning, sneezing, hiccups, change of skin colour, tremor and straightening of limbs (13,14).

Infants born prematurely are the ones most exposed to the effects of an adverse sound environment. They are deprived of the sound stimulation of the intrauterine environment and, for many weeks, are exposed to the loud and unpredictable environment of the neonatal intensive care unit. Exposure to noise is the cause of many homeostatic disorders in a child (6).

## Objective

The aim of the study was to analyse the sound environment and the range of sound levels recorded in the newborn's environment immediately after birth.

## Materials and methods

The research method used in the work was direct observation. The research material was collected using an authored observation sheet designed for the purposes of the study. The observation was combined with the recording of sound intensity, expressed in decibels, using measuring equipment. To carry out the measurements, certified environmental factor measuring devices (environmental meters- Extech EN 300) were used. Prior to the commencement of the test, the devices were calibrated in an accredited laboratory of a confirmed quality by the Polish Centre for Accreditation, in which the calibration was in accordance with the requirements of International Organization for Standardization/International Electrotechnical Commission (ISO/IEC) 17025: 2005. The verification of the measurements was performed on the task plane (at the height of the newborn's head).

A total of 304 randomly selected observations were carried out in the delivery room at 11 obstetric facilities in Warsaw. The study was conducted after obtaining the consent of the hospital management. The sound recordings were made in the period from January 2016 to December 2017. The criterion for inclusion in the study was the good general condition of the newborn (obtaining ³8 points in the first and fifth minutes of life on the appearance, pulse, grimace, activity and respiration [APGAR] score). Observation using measuring apparatus was carried out during the newborn baby's first hour of life. During the implementation of the study, national and international standards of ethics in human research were observed. AP5 IStatistical analysis was performed using the Statistica 13 programme. Quantitative variables were presented as minimum, maximum, mean value and standard deviation, as well as the median and quartile range (values of the first and third quartiles). Spearman's correlation coefficient was used to analyse the data.

## Results

The study was conducted in 11 obstetric hospitals with different reference levels, comprising a total of 304 observations. The percentage comparison depending on the institution participating in the study is presented in Table 1.

**Table 1:** Number of deliveries observed

	**Hospital A**	**Hospital B**	**Hospital C**	**Hospital D**	**Hospital E**	**Hospital F**	**Hospital G**	**Hospital H**	**Hospital I**	**Hospital J**	**Hospital K**	**Total**
**N**	**81**	**51**	**48**	**30**	**24**	**14**	**14**	**12**	**11**	**11**	**8**	**304**
%	26.64	16.78	15.79	9.87	7.89	4.61	4.61	3.95	3.62	3.62	2.63	100.00

Among the newborns examined, the largest group comprised full-term newborns (91.45%). The group of premature newborns accounted for 8.55%. The mean week by foetal age was 39.09 ± 1.20 weeks, with median of 39 weeks. The week of completion of pregnancy in the study group was between 35-0/7 and 42-0/7 weeks. The general condition of all the naturally delivered newborns who were analysed was assessed as good; these newborns obtained 8–10 points on the Apgar scale in both the first and the fifth minutes of life. The average level of sound intensity measured in decibels was 58.03 ± 7.66. Sound intensity ranged from 40.30 dB to 78.50 dB, and the median was 58.55 dB (Table 2).

**Table 2:** Sound level

	**N**	**Minimum**	**Maximum**	**Mean**	**SD**	**Median**	**Q1**	**Q3**
Sound level, dB Natężenie dźwięku	304	40.30	78.50	58.03	7.66	58.55	52.00	63.35

Among the hospitals analysed, the highest recorded average sound level in one of the facilities was 64.41 ± 4.91 dB (median: 66.40 dB). The lowest average sound level in another location was in the range 54.79 ± 6.55 dB (median: 53.00 dB). The minimum values ranged from 40.30 dB to 56.00 dB. The maximum values were 68.20 ± 78.50 decibels (Table 3).

**Table 3:** Individual hospitals: sound level

	**N**	**Minimum**	**Maximum**	**Mean**	**SD**	**Median**	**Q1**	**Q3**
Hospital A	81	43.10	68.20	54.79	6.55	53.00	49.90	60.30
Hospital B	51	45.00	78.30	58.54	7.32	59.00	52.50	62.00
Hospital C	48	44.90	70.00	58.07	5.58	58.65	53.40	62.35
Hospital D	30	41.00	72.00	60,80	7.64	61.50	55.00	66.00
Hospital E	24	40.30	78.00	59.37	10.48	59.50	51.35	67.00
Hospital F	14	45.00	70.10	57.26	7.14	56.50	54.00	59.40
Hospital G	14	48.00	71.50	59.71	6.20	61.25	55.80	64.00
Hospital H	12	43.40	78.00	61.02	12.58	66.00	49.50	69.15
Hospital I	11	46.00	68.40	55.32	5.91	53.50	51.60	58.40
Hospital J	11	56.00	72.00	64.41	4.91	66.40	59.40	67.10
Hospital K	8	48.50	78.50	61.92	8.92	59.50	57.85	66.85

The most common sources of noise observed in the delivery room were staff conversations (80.26%), conversations in the corridor or in the adjacent room (20.07%), dynamic closing of drawers (14.80%), opening of single-use materials (10.86%), crying baby (5.59%), moving equipment (4.93%) and having another delivery in the same room (2.96%). In addition to the mother, 1–12 people were in the delivery room, and the median was 2.00 (Tables 4 and 5).

**Table 4:** Number of people in the delivery room

	**N**	**Minimum**	**Maximum**	**Mean**	**SD**	**Median**	**Q1**	**Q3**
Number of people	304	1.00	12.00	2,63	1.88	2.00	1.00	4.00

**Table 5:** Individual hospitals: number of people in the delivery room

	**N**	**Minimum**	**Maximum**	**Mean**	**SD**	**Median**	**Q1**	**Q3**
Hospital A	81	1.00	4.00	1.51	0.81	1.00	1.00	2.00
Hospital B	51	1.00	8.00	2.31	1.35	2.00	2.00	3.00
Hospital C	48	1.00	4.00	1.85	0.95	2.00	1.00	2.00
Hospital D	30	1.00	10.00	3.97	2.24	4.00	3.00	5.00
Hospital E	24	1.00	10.00	4.42	2.48	5.00	2.00	6.00
Hospital F	14	1.00	5.00	2.64	1.45	2.00	2.00	4.00
Hospital G	14	1.00	6.00	3.79	1.31	4.00	3.00	5.00
Hospital H	12	1.00	12.00	4.17	3.19	3.00	3.00	4.00
Hospital I	11	1.00	4.00	1.82	1.40	1.00	1.00	4.00
Hospital J	11	3.00	6.00	4.91	0.94	5.00	4.00	6.00
Hospital K	8	2.00	6.00	4.00	1.41	4.00	3.00	5.00

A statistically significant relationship between the number of people in the delivery room and sound intensity was demonstrated. The number of people positively correlated with the average sound level (*R*=0.520, *p*<0.001). The more people there were in the place of delivery, the greater was the volume of sound noted (Table 6).

**Table 6:** Impact of the number of people on sound intensity

	**N**	**R^a^**	**p^b^**
Impact of the number of people on sound intensity	304	0.520	<0.001

a *R – correlation coefficient*;

b *p – significance level*

There was no statistically significant difference between the sound intensity level in the delivery room and the newborn's maturity (*p*=0.430).

## Discussion

Current global recommendations limit the level of sound emission in neonatal wards to 45 dB. Based on the study, it was found that the average sound level in the delivery room exceeded the recommended maximum permissible noise level; the mean sound level in the first hour of life was 58.02 ± 7.77 dB (median: 58.55 dB). Maximum values were 68.20–78.50 dB. Different sources indicate that the maximum allowable and recommended level of 40–45 dB is exceeded in many neonatological intensive care units (15,16). This is particularly worrying because studies have shown that noise acts as a stressor for both patients and staff (17), which can lead to many negative health effects. For example, noise acts as a permanent stimulator of the sympathetic nervous system, resulting in an increase in heart rate and blood pressure (18). Oliveira et al. (1918) indicate that sudden loud sounds from 70 dB to 75 dB may cause agitation in and crying of a child, leading to an increase in intracranial pressure, changes in heart rate and breathing, as well as reduced percutaneous oxygen saturation, which may result in changes in brain tissue perfusion.

A newborn baby may be exposed to various sources of noise after birth. In studies by Chen and Chang (2019), it was observed that the most common source of noise were conversations conducted by staff. These results are similar to the results of tests in neonatal intensive care units (2019, 210). Research by Boehm and Morast (221) shows that the main source of noise in neonatal intensive care units is most often the working equipment and the activity of medical personnel. Medical staff generate unfavourable sounds, by means such as loud conversations, which – according to the authors – often translates into a sound level of 55–67 dB. Activities such as dynamic closing of drawers and cabinets, opening of disposable equipment or disconnection of medical gases from the socket also contribute to the creation of high sound levels of about 89 dB (11). In our own study, it was observed that the most common source of noise in the delivery room was staff conversations.

It has been shown that there is a positive correlation between the level of sound intensity and the number of people (*R*=0.520, *p*<0.001). The more people there were around the newborn, the higher was the volume. The results of the study showed that the behaviour of medical staff was of great importance in determining the acoustic profile of the rooms tested. It is worth noting that noise from the corridor or adjacent room was the second most common source, which underlines the importance of a behavioural factor. Due to the fact that healthcare activities are the main source of noise, it is important to pay attention to the need to reduce the number of staff. The demonstration, in many studies, of the adverse effect of noise on newborn development leads to the search for solutions to reduce the exposure of the newborn to noise (23). It is necessary to implement recommendations to reduce bad habits and to require the application of good practices. Noise reduction can improve the working environment of the medical team and, thus, its clinical performance (24).

To reduce the child's anxiety and negative sensations, it would be best to remain silent. The only sound that keeps a child safe is the voice of the parents. To facilitate the mother's first contact with the child, which is crucial for bonding, it is important to place the newborn on the mother's abdomen immediately after birth (25,26). Recommendations for reducing the risk of exposure to noise include the use of equipment that generates a minimum level of noise. In order to improve the conditions of the sound environment, it would be necessary to remove objects that generate constant sounds (e.g. radio). Conversations in the room where the newborn is should be quiet, preferably not directly at the newborn. Within the department, cell phones should be muted; footwear worn by staff should have soft soles. It is important to properly operate the apparatus, open single-use packaging silently, lower the levels of alarms and buttons emitting sound to the necessary minimum and immediately react to the alarm signalling (27).

In intensive care units, in order to reduce noise levels, the so-called “Quiet Time” is observed. This is the time during which staff activity is minimised, and patients have uninterrupted time. The lighting is reduced, phone signals are muted, staff conversations are limited and diagnostic tests and all procedures are carried out at a different time, if possible (28,29). This principle of “quiet time” could also be adopted for the first hour of life of a newborn whose general condition and the condition of the mother allow “skin to skin” contact. Sounds known from intrauterine life, primarily the mother's voice and her heartbeat, undisturbed by other stimuli, will play a protective role in the newborn's entry into a new environment. In order to protect the newborn from noise, continuous monitoring of the sound level in his/her surroundings is recommended. A warning system is used in the form of sound monitoring devices. These devices are designed to create awareness among staff and parents by monitoring the sound level in hospital wards (30). Such a warning system could also be used in delivery rooms to increase team awareness.

## Conclusions

The average sound level in the delivery room exceeded the recommended standards.

The noise was mainly caused by the staff's activity and positively correlated with the number of people in the newborn's surroundings.

It is important to educate staff to minimise sources of noise, provide better working conditions for the team and ensure patient safety.
